# Hazardous waste and health impact: a systematic review of the scientific literature

**DOI:** 10.1186/s12940-017-0311-8

**Published:** 2017-10-11

**Authors:** L. Fazzo, F. Minichilli, M. Santoro, A. Ceccarini, M. Della Seta, F. Bianchi, P. Comba, M. Martuzzi

**Affiliations:** 1 Department of Environment and Health, Unit of Environmental and Social Epidemiology, Istituto Superiore di Sanità, viale Regina Elena 299, 00161 Rome, Italy; 2 Institute of Clinical Physiology, Unit of Environmental epidemiology and disease registries, National Research Council, Via Giuseppe Moruzzi 1, 56124 Pisa, Italy; 3 Documentation Service, Istituto Superiore di Sanità, viale Regina Elena 299, 00161 Rome, Italy; 4 Centre for Environment and Health, World Health Organization - Regional Office for Europe, Platz der Vereinten Nationen 1, D-53113 Bonn, Germany

**Keywords:** Waste, Hazardous waste, Health, Cancer, Disease, Congenital anomalies, Review

## Abstract

Waste is part of the agenda of the European Environment and Health Process and included among the topics of the Sixth Ministerial Conference on Environment and Health. Disposal and management of hazardous waste are worldwide challenges. We performed a systematic review to evaluate the evidence of the health impact of hazardous waste exposure, applying transparent and a priori defined methods. The following five steps, based on pre-defined systematic criteria, were applied. 1. Specify the research question, in terms of “Population-Exposure-Comparators-Outcomes” (PECO). Population: people living near hazardous waste sites; Exposure: exposure to hazardous waste; Comparators: all comparators; Outcomes: all diseases/health disorders. 2. Carry out the literature search, in Medline and EMBASE. 3. Select studies for inclusion: original epidemiological studies, published between 1999 and 2015, on populations residentially exposed to hazardous waste. 4. Assess the quality of selected studies, taking into account study design, exposure and outcome assessment, confounding control. 5. Rate the confidence in the body of evidence for each outcome taking into account the reliability of each study, the strength of the association and concordance of results.

Fifty-seven papers of epidemiological investigations on the health status of populations living near hazardous waste sites were selected for the evidence evaluation. The association between 95 health outcomes (diseases and disorders) and residential exposure to hazardous waste sites was evaluated. Health effects of residential hazardous waste exposure, previously partially unrecognized, were highlighted. Sufficient evidence was found of association between exposure to oil industry waste that releases high concentrations of hydrogen sulphide and acute symptoms. The evidence of causal relationship with hazardous waste was defined as limited for: liver, bladder, breast and testis cancers, non-Hodgkin lymphoma, asthma, congenital anomalies overall and anomalies of the neural tube, urogenital, connective and musculoskeletal systems, low birth weight and pre-term birth; evidence was defined as inadequate for the other health outcomes. The results, although not conclusive, provide indications that more effective public health policies on hazardous waste management are urgently needed. International, national and local authorities should oppose and eliminate poor, outdated and illegal practices of waste disposal, including illegal transboundary trade, and increase support regulation and its enforcement.

**Electronic supplementary material:**

The online version of this article (10.1186/s12940-017-0311-8) contains supplementary material, which is available to authorized users.

## Background

This paper presents a systematic review of the available literature on health effects of the residence in the vicinity of hazardous waste sites. Our aim was to evaluate the evidence of the association between exposure to hazardous waste and health outcomes.

The term “hazardous” waste is variously applied in different countries, loosely defining non-household waste that includes hazardous chemicals. In our search literature, we included the terms “hazardous”, “toxic”, “industrial” waste, excluding the papers about municipal landfills, which have no records of hazardous materials, incinerators, e-waste and radioactive waste disposals. The present review does not consider occupational studies.

Waste, and in particular hazardous waste, is one of the priority areas for the Member States of the World Health Organization (WHO) Regional Office for Europe and was in the agenda of the Sixth Ministerial Conference on Environment and Health [1].

Disposal and management of waste are world-wide problems. Poor, outdated and illegal practices of urban and hazardous waste disposal affect local communities virtually in all countries; this includes illegal transboundary trade, mostly from industrialized countries [2]. The burden of diseases of waste-related exposures in middle-low income countries is increasing and not sufficiently recognized [3].

Several investigations indicate poor and illegal waste management as the most important world-wide cause of contamination of soil and groundwater.

In January 2007, the US Environmental Protection Agency’s National Priority List (NPL) included 1240 hazardous waste sites, comprising 157 federal facilities. The Environmental Protection Agency (EPA) estimated 41 million people were living within a 4-mile radius of an NPL site in 2007. Waste storage/treatment/disposal are the main activities in the 1684 present or past NPL sites across the country (31.5%), followed by manufacturing and industries (30.8%) [4].

In Europe, in 2014, 342,000 contaminated sites were identified (5.7 per 10,000 inhabitants). On the basis of the data provided by 33 countries, in 2011 the activities which contributed most to soil and groundwater contamination were waste disposal, including municipal and industrial waste (about 38% of the sites), and industrial and commercial activities (mining, oil extraction and production, power plants - about 34% of the contaminated sites) [5].

This type of data is less frequently available in middle-low income countries. In seven Asian countries, 679 areas were identified as contaminated by hazardous waste. Of these, 169 sites were polluted by lead resulting in an estimated 245,949 0–4 years old children exposed to lead. The estimated levels of exposure might be sufficient to generate acute and chronic adverse effects, such as a decrease in Intelligence Quotient (IQ) [6]. Chatman-Stephens and colleagues analyzed 373 hazardous waste sites in three Asian countries (India, Indonesia, Philippines) and estimated approximately 9 million people to be at risk; adding another estimated 43 million people at risk from unscreened sites to the exposed population, 4 million DALYs (disability-adjusted life years) associated with hazardous waste sites were estimated as the impact [7].

In Africa, where WHO estimates that 1/3 of the burden of disease is attributable to environmental risk factors [8], hazardous waste have been included among the first three main such factors [9]; domestic and hazardous waste management is of particular concern [10]. In most African cities, less than 20% of urban waste is disposed of in landfills. The remaining waste ends up in illegal dumps [11]. Africa is also one of the main destinations of illegal transboundary trade of urban and hazardous waste from industrialized countries [2].

In this context, waste from end-of-life electrical and electronic equipment, so called “e-waste”, is especially important. E-waste contains recognized hazardous substances that may be directly released or generated after disposal or during the recycling process. Unsafe recycling techniques in middle-low income African and Asian countries, where 75% and 80% of respectively EU- and USA-produced total e-waste is illegaly exported, involve high risks, primarly for workers, who are often children and women [12].

In recent years, investigations reporting a wide spectrum of health risks for local populations living in the areas surrounding hazardous waste dumping sites have been published. Still, an up-to-date evaluation of the evidence of the association between adverse health effects and hazardous waste is not available.

In 2000, a review of hazardous waste reported that the evidence of a causal relationship with cancers “is still weak”, especially with regard to specific cancers reported in more than one study: leukemia, bladder, lung and stomach cancers [13]. A relationship was suggested with adverse pregnancy outcomes, i.e. low birth weight, total birth defects and cardiac, musculoskeletal and central nervous system defects. However, the authors considered that the studies were still too few to draw conclusions regarding causality.

In 2007, a WHO report on waste and health concluded: “Despite the methodological limitations, the scientific literature on the health effects of landfills provides some indication of the association between residing near a landfill site and adverse health effects. The evidence, somewhat stronger for reproductive outcomes than for cancer, is not sufficient to establish the causality of the association. However, in consideration of the large proportion of population potentially exposed to landfills in many European countries and of the low power of the studies to find a real risk, the potential health implications cannot be dismissed”. The Report deals with landfills at large and not specifically with hazardous waste management; some case-studies, though, address this issue and recommendations about study design are provided [14].

A review on cancer epidemiology studies of populations resident close to toxic waste sites concluded that available studies provided valid hypotheses, but cannot determine whether residence near toxic waste sites causes an increased cancer risk [15].

A subsequent review of the literature on health impact of municipal waste management assessed the evidence of association with urban waste landfills as limited for total, neural tube and genitourinary birth defects, and low birth weight [16]. Consistent conclusions were reached by Mattiello and colleagues [17].

In this context, a systematic review is presented to evaluate the evidence of health impact of hazardous waste exposure, applying a priori defined criteria.

## Methods

As recently recommended by authoritative agencies, such as the US EPA and WHO [18–21], established methods and selection criteria defined a priori were applied.

A five-step process, as described by Woodruff, was followed: 1. Specify the research question; 2. Carry out the literature search, specifying the search strategy with sufficient detail so that it can be reproduced; 3. Select studies for inclusion, analyzing their compliance with a priori defined criteria; 4. Assess the quality of individual selected studies; 5. Rate the confidence in the body of evidence for each outcome [20]. In detail:

1. The research question was formulated in terms of “Population-Exposure-Comparators-Outcomes” (PECO): Population: people living near hazardous waste sites; Exposure: exposure to hazardous waste; Comparators: all comparators; Outcomes: all diseases/health disorders.
2. Search strategy: the search was conducted in Medline and EMBASE, on STN International (Information Service for research and patent information: stnk.fiz-karlsruhe.de), using both text-words (Table 1) and descriptors (Table 2) based on the above PECO question. Boolean operators were used to combine exposure and outcome terms, as reported in the tables. The search was limited to the articles published in the 1999–2015 period, i.e. after those considered in the review by Vrijheid et al. [13].
3. Select studies for inclusion. Original epidemiological studies on populations residentially exposed to hazardous waste were considered; the criteria for inclusion of articles in the review were defined a priori. Exclusion criteria based on types of waste were defined: urban waste (landfills/incinerators), e-waste and radioactive waste; the latter two because of their peculiarities (potentially released agents and affected sub-populations). Reviews and economic evaluations were not included in the evidence evaluation, but considered subsequently for discussion purposes. Biomonitoring and toxicological studies were not considered as part of the main body of primary evidence, but were retained for interpreting and evaluating the findings of epidemiological investigations.

**Table 1:** Text-word searching in Medline and EMBASE

Search	Text words
#1 Exposure	(INDUSTR? OR ILLEGAL OR HAZARDOUS OR TOXIC) (W) (WAST? OR LANDFILL OR DUMP?)
#2 Health outcomes	(RESPIRATORY OR CARDIOVASCULAR OR URINARY OR KIDNEY) OR (ADVERSE EFFECT OR HEALTH EFFECT OR HEALTH IMPACT) OR (CANCER OR TUMOR OR NEOPLASM) OR (CONGENITAL OR REPRODUCT? OR BIRTH OR NEONATAL) OR (BIRTH? OR REPROD? OR TERATO?) OR (DIABETE? OR THYROID?) OR (ACUTE EFFECT) OR (ACUTE TOXIC?)
#3 Biomonitoring	(BIOMONITOR? OR BIO(W) MONITOR?)
#4	#1 AND #2 AND #3

**Table 2:** MeSH (Medline) and EMtree (EMBASE) descriptors used in search strategy

Search	
	MeSH descriptors
#1 Exposure	(HAZARDOUS WASTE + NT/CT) AND ((ENVIRONMENTAL HEALTH + NT/CT) OR ENVIRONMENTAL EXPOSURE + NT/CT))
#2 Health outcomes	(EPIDEMIOLOGY/CT)^a^ OR (MORTALITY/CT)^b^
#3	#1 AND #2
	EMtree descriptors
#1 Exposure	*HAZARDOUS WASTE + NT/CT
#2 Health outcomes	EPIDEMIOLOGY + NT/CT
#3	#1 AND #2

^a^Epidemiology: used with human and veterinary diseases for the distribution of disease, factors which cause disease, and the attributes of disease in defined populations; includes incidence, frequency, prevalence, endemic and epidemic outbreaks; also surveys and estimates of morbidity in geographic areas and in specified populations. Used also with geographical headings for the location of epidemiologic aspects of a disease. Excludes mortality for which “mortality” is used. (National Library of Medicine)

^b^Mortality: Used with human and veterinary diseases for mortality statistics. For deaths resulting from various procedures statistically but for a death resulting in a specific case, use FATAL OUTCOME, not /mortality. (National Library of Medicine)

Each study identified in the initial literature search was assessed for inclusion independently by two investigators. Studies were included or excluded upon concordant assessment; a third investigator’s opinion was used in case of discordant assessment.

4. Assess the quality of individual studies. The full team of investigators established the reliability of each study. This step involved an evaluation of the possible role of chance (random error) and bias (systematic error), arising from flawed exposure and outcome assessment, confounding factors or study design leading to selection bias. A qualitative rating of these items (exposure and outcome assessment, confounding control) was included in the assessment and is reported in tables, with a notation ranging from “- -”to “+ +”. The overall assessment was not univocally determined by these ratings, but reflected a final expert judgement of each paper’s quality.

This evaluation was performed individually by one investigator per paper and, subsequently, discussed by the overall group. The reliability of each study was defined in 5 classes, from low (1) to high (5).

5. Rate the strength of the body of evidence, for each outcome. The evaluation of the evidence of association between each health outcome and hazardous waste exposure was estimated taking into account the reliability of each study, the magnitude and the accuracy of the estimated association, and the concordance between study findings. The evidence was rated in three grades: Sufficient/Limited/Inadequate, partly derived from the approach used by the International Agency for Research on Cancer (IARC) Monographs [22], but specifically defined, as follows.

Sufficient: More than one study of high or moderate/high quality (rated 5–4) report positive findings with strong (high values of relative risk) and precise, overall consistent association. Alternative explanations, in particular the role of random variability, bias, confounding factors, can be reasonably excluded. The force of association, considerations on dose-response relationship, time coherence and biological plausibility further support causality.

Limited: More than one study of high or moderate/high quality (rated 5–4) report positive findings with strong (high values of relative risk) and precise association. Among the concurring different risk estimates, the results of higher quality studies was given higher weight. A role of random variability, bias and confounding factors may not be completely excluded.

Inadequate: Less than two studies of moderate or higher quality rate (rated 5–3) report findings of risk in excess; or, there are two or more studies of moderate/high quality, but the results in excess are not consistent and/or the associations are weak and inaccurate.

## Results

Two thousand five hundred ninety three records were retrieved from MedLine and EMBASE text-word and descriptor searching; 913 of these were excluded because published before 1999.

**Fig. 1:**
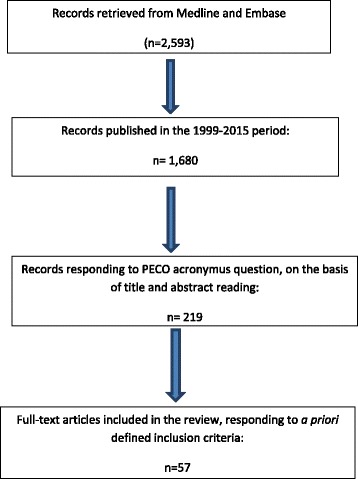
Flow chart showing the literature search and screening process

Of the remaining 1680 records, 1461 were excluded on the basis of title and abstract, because not responding to the a priori defined PECO question. The abstracts of the resulting 219 papers were then evaluated independently by two investigators on the basis of the inclusion criteria. The results of this first evaluation were reviewed by the full team of investigators. 167 papers of epidemiological investigations on populations living near hazardous waste sites were selected. Following a review of the full-text of each study, 110 were considered not relevant and were excluded. 57 articles were eventually selected and underwent the evaluation of the strength of evidence (Fig. 1).

Most of the selected studies reported more than one health outcome. Altogether, the association between 95 health outcomes (diseases and disorders) and residential exposure to hazardous waste sites was reported on.

Additional file 1: Table S1, Additional file 2: Table S2 and Additional file 3: Table S3 report, for each outcome, the studies’ characteristics, the qualitative rating of risk of bias, a reliability assessment and the overall evidence evaluation.

As a result, the evidence of the causal association between hazardous waste related exposures and chronic and reproductive health outcomes was evaluated as: limited, for liver, breast, testis and bladder cancers, non-Hodgkin lymphoma and asthma (Additional file 1: Table S1), overall and specific congenital anomalies (urogenital, neural tube, musculoskeletal and connective system), low birth weight and pre-term birth (Additional file 2: Table S2); inadequate, for all the other health outcomes.

Studies on acute effects in populations living near illegal dump sites in Abidjan [23, 24] provided sufficient evidence of association between exposure to oil industry waste and general and neurological acute symptoms, and specifically otolaryngological, respiratory, digestive and dermatological symptoms (Additional file 3: Table S3).

## Discussion

The present review considers literature pertaining sites where hazardous waste was handled without control or suitable environmental management. These practices take place both in high and low-middle income countries. However, they occur more frequently in the latter, through illegal dumping of hazardous waste, or when waste dumping took place prior to the enforcement of environmental regulations, or in countries where there is no regulation. In some cases, hazardous waste can be unknowingly present in landfills.

Rigourous methodology for systematic and a priori criteria-based reviews was used. The application of criteria of “reliability” based on risk of bias to observational studies requires some consideration. In order to evaluate the strength of study results, and their risk of bias, exposure and outcome assessment methods, confounding control and study design were considered. Most of the selected studies were ecological, a design which is generally regarded as weaker than individual-level studies, because of the so-called “ecological fallacy” (i.e., interpreting associations at the aggregated level as causality at the individual level) [25]. Ecological studies, however, have played a major role in the investigation of etiological associations of public health importance [22, 25].

The reliability of the majority of the studies were considered “moderate”, primarily due to the limitations of exposure assessment based on the residence at outcome observation time. Data about the contaminants present in the waste site at the time of the study and in the surrounding residential environment was rarely available. When it was, a higher weight was assigned to the study.

Furthermore, the definition of hazardous waste limits comparability between studies. Hazardous waste includes a broad range of contaminants affecting different environmental matrices and involving several routes of exposure, depending on types of waste and hydrogeological and meteorological factors. These limitations tend to underestimate, rather than overestimate, the magnitude of health effects, thus diluting the evidence for specific types of hazardous waste exposure.

The evidence of the association between general and neurological acute symptoms, in particular those regarding otolaryngological, respiratory and digestive systems and the skin, and exposure to oil industry waste releasing high concentrations of hydrogen sulphide was evaluated as sufficient. This evaluation was based on two cross-sectional studies performed in the population resident in Abidjan near the sites where approximately 500 tons of hazardous waste was illegaly dumped and subsequently emitted hydrogen sulphid into the air [23, 24]. The two studies were evaluated respectively as being of high and moderate/high quality (rated 5–4). They consistently reported elevated relative risks, with good accuracy. Furthermore, strength of association, presence of a dose-response relationship, time coherence and biological plausibility support causality. Alternative explanations, in particular the role of random variability, systematic bias and confounding factors, can be reasonably excluded.

Contaminants emitted or released by hazardous waste might play a role for the occurrence of diseases with multi-factor etiology, however evidence of an association was limited, as in the case of liver cancer. In the above-mentioned review of municipal waste management, inadequate evidence was attributed to the association between liver cancer and landfills [16]. B and C hepatitis viruses, alcohol consumption, tobacco smoking and aflatoxins are the main ascertained risk factors for liver cancer. Exposure to vinyl chloride and 1,2-dichloropropane was defined by IARC as associated with hepatic cancer with sufficient evidence. The evidence regarding the association of arsenic and its inorganic compounds, DDT, dichloromethane and trichloroethylene was defined as limited [26]. Even if 80–95% of hepatocellular carcinomas at the global level are associated with B or C hepatitis chronic viral infection [27], an interaction between chemicals and the other risk factors has been suggested, i.e. occupational exposure to vinyl chloride monomer (VCM) and hepatitis B virus infection [28, 29].

Hepatotoxic chemical agents can be both naturally occurring and synthetic; the latter comprise metals, aromatic and halogenated hydrocarbons, chlorinated aromatic and nitro compounds that can, to various extents, be present in or released by hazardous waste [29]; hepatotoxicity can be related to hepatic carcinogenicity.

Our evaluation on liver cancer was based on the results of ten articles; some of them reported the presence of organic chlorinated compounds, such as vinyl chloride [30] and beta-hexachlorocyclohexane, β-HCH [31], and of heavy metals, including arsenic [31]. Other studies [32, 33] regarded areas in which biomonitoring detected presence of dioxins in breast milk of women [34]. In the latter studies the synergistic effects between different risk factors, including B hepatitis virus and hazardous waste contaminants, may be reasonably hypothesized.

We attributed limited evidence to the association between bladder cancer and hazardous waste on the basis of ten articles. The previous review by Porta defined the association between bladder cancer and urban waste disposals as limited [16]; another review found bladder cancer among those reported in excess in more than one study [13]. A number of agents were defined by IARC as associated with bladder cancer with sufficient evidence, including tobacco smoke, arsenic and its compunds, and occupational exposures occurring in industries involving the production and/or use of aluminum, auramine, magenta, rubber, and paint; evidence was classified as limited for occupational exposure of hairdressers and barbers, coal-tar pitch, printing processes and textile manufacturing, and, among chemical agents, for specific chloro-compounds, i.e. 4-chloro-ortho-toluidine and tetrachloroethylene [26]. A specific association between bladder cancer and benzo(a)pyrene was reported elsewhere [35].

Some of the ten articles on bladder cancer considered in the present review reported the presence of contaminants associated with bladder cancer in the study areas. Heavy metals in soil and groundwater, and β-HCH in blood of some residents were reported in the Sacco river areas [31]; hexachlorocyclohexanes, benzylchlorides, organic sulfur compounds, chlorobenzenes, and sodium sulfide/sulfhydrates polluted the Love Canal site [36], where a biomonitoring study reported also trichlorobenzene and dichlorobenzene contamination [37]. Dioxins were detected in biomonitoring studies in Campania waste sites [34] located in towns where increased bladder cancer risk was reported [32, 33].

Nine articles included in the present review determined the evaluation of limited evidence for the association between non-Hodgkin lymphoma (NHL) and hazardous waste: excess risks were reported in areas contaminated by organic chloro compounds, including vinyl chloride [30] and β-HCH [31], heavy metals [31] and, in Superfund sites, in areas with benzene-emitting hazardous waste sites [38]. The main known risk factors of NHL, associated with sufficient evidence according to IARC, are viral factors (Epstein-Barr virus, hepatitis C virus, HIV type1). Several chemical agents were defined as risk factors for NHL with limited evidence: benzene, ethylene oxide, 2,3,7,8-tetrachlorodibenzo-para-dioxin (TCDD), PCBs, tetrachloroethylene, trichloroethylene, polychlorophenols and their sodium salts [39].

For breast and testis cancers, the evidence was evaluated taking into account similarities in their biological characteristics. Limited evidence attributed to breast and testis cancers is based on the etiological plausibility of their relationship with endocrine disruptors (EDCs). This evaluation is corroborated by the results found for urogenital tract defects. A role of exposure to EDCs was hypothesized in incidence of breast and testis cancers and urogenital tract anomalies [40]: positive results for these outcomes in the selected studies and the possible presence of these contaminants in hazardous waste sites support the hypothesis of a causal relationship. A European case-control study on occupational exposure to endocrine disrupting chemicals in male breast cancer suggested a possible role of occupational exposures to oil and petroleum solvents in motor vehicle mechanics and to chemicals such as alkylphenolic compounds [41]. IARC defined as limited the association between breast cancer and the exposute to dioxins, ethylene oxide and PCBs [26].

In our review, five articles on breast cancer were considered, including the above-mentioned studies on areas with documented contamination [31, 32, 36] and an article on populations living in zip code areas near Superfund sites polluted by volatile organic compounds (including chloroethenes, chloroethanes, chloromethanes, chlorobenzenes, benzene, toluene, ethyl benzene, xylene) [42].

For testis cancer, IARC assessed limited evidence for DDT, diethylstilbestrol (exposure in utero), N,N-dimethylformamide, perfluorooctanoic acid; no agents were defined as associated with testis cancer with sufficient evidence [26]. An etiological role of exposure to endocrine disruptors in the onset of testis cancer was hypothesized [43] and suggested in some reviews [44, 45]. A possible association between endocrine disruptors and testicular cancer could be explained by the Testicular Dysgenesis Syndrome, reflecting a hormonal imbalance due to environmental or life-style factors during early fetal development [40, 46, 47] and during puberty due to the ingestion of contaminated milk [47].

Finally, evidence of hazardous waste effects on asthma and adverse reproductive effects was considered limited. We based the evaluation for asthma on five studies considering, in particular, populations (both adults and 0–14 year old children) living near hazardous waste sites with emission of POPs (dioxins/furans, PCB, chlorinated pesticides) [48, 49], heavy metals and β-HCH [31] and other organic compounds [50].

In 2006, WHO suggested that airborne particulate matter contributes to the exacerbation of asthma based on “considerable” evidence. Atmospheric particles, including acid aerosols derived from sulfur dioxide emissions, have been linked with worsening of symptoms, reduction in lung function, increased hospital admissions for asthma and increased use of medication. The evidence that exposure to certain kinds of particulate matter (PM), such as diesel exhausts, may contribute to causing asthma in susceptible subjects has been defined as “suggestive” [51]. A Report of the US Department of Health and Human Services defined the evidence of the causal role of active and second-hand smoke, in the incidence and exacerbation of asthma as sufficient or suggestive in different sub-groups of the population [52]. Therefore, it is plausible that hazardous waste emitting air pollutants might cause asthma in susceptible subjects or exacerbate asthma cases.

Diabetes and infectious respiratory diseases, for which the evidence of association with hazardous waste was evaluated as inadequate, deserve some considerations. Two studies included in our review reported consistent findings of an increased risk for diabetes. One study was carried out in an area with heavy metals in the soil and groundwater and β-HCH in dairy products, forage, soils and in blood samples of residents [31], and the other in a Superfund site with contamination by Persistent Organic Pollutants, POPs (PCBs, dioxins/furans, or chlorinated and persistent pesticides) [53]. Taking into account the high prevalence of the disease and the need to thoroughly assess the role of its many ascertained risk factors, the evaluation of the role of environmental exposures is challenging. A recent systematic review indicated a positive association between diabetes and serum concentrations of several pollutants (such as polychlorinated dibenzodioxins and dibenzofurans, PCBs, and several organochlorine pesticides) [54]. Type 2 diabetes has recently been recognized as a risk modifier for PM health effects, enhancing mortality risks associated with PM exposure [51]. The evidence of a causal association between the risk of diabetes and active smoking was defined as sufficient, with a positive dose-response relationship with the number of cigarettes smoked [52].

We evaluated as inadequate the evidence of the association of hazardous waste with “acute infections of the respiratory system, pneumonia, influenza” because of the lack of consistency between studies. However, consistent results of increased risk were reported near waste sites with air emissions of POPs [48, 49]. Taking into account the available evidence on health effects of air pollution [51], these results require further confirmation, notably in populations living near hazardous waste that emits air pollutants.

Regarding congenital anomalies (CAs), the evidence of association to hazardous waste was evaluated as limited for overall and some subgroups of CAs (i.e. urogenital, connective and musculoskeletal system and neural tube anomalies). These findings are consistent with previous reviews [13, 16, 17]. Our update confirms some limitations mainly due to the small number of studies and weak exposure assessment. As CAs are short-term latency outcomes, they are important for investigating short-term effects in polluted areas. The etiology of CA is multi-factorial, with an important role of genetic factors, and a recognized interaction between genetic and environmental factors [55] and epigenetic mechanisms; this topic deserves further investigation in order to better clarify the etiological pattern.

Since CAs are relatively rare events, affecting about 2–3% of births, groups including several specific anomalies are usually investigated in epidemiological studies. Despite this, the reported risk estimates are often not accurate. Furthermore, the interpretation of results regarding groups of CAs with different causes is difficult and requires caution [56]. Two of the reviewed studies investigated the association between exposure to hazardous waste and hypospadias. This association is of interest since the vast majority of cases seem to have multi-factorial aetiology, with many genetic and environmental factors playing a role, in particular endocrine-disrupting chemicals [57–59]. Our results are in agreement with a recent review claiming that the literature is currently poor and does not support a conclusive judgment on environmental contaminants and congenital anomalies in the general population [60].

Among the five studies considered for pre-term birth evaluation only two were evaluated as high quality, and although the estimated risks were above unity for all the studies, none reached statistical significance, except in the study of Love Canal residents, who had a significant risk of pre-term birth compared to New York State reference rates. The overall results on pre-term birth, however, could reflect the studies’ low statistical power and do not warrant an evaluation of evidence higher than limited.

The studies on low birth weight (LBW) were more numerous; among the nine studies considered, one was of high quality, reporting no significantly increased risks [61], and two were of moderate-high quality and reported statistically significant excess of risk [62, 63]. Although the body of evidence is richer than for pre-term birth, we believe that it is still not enough to upgrade the evaluation of limited evidence previously assigned by Porta (2009) [16] and Mattiello (2013) [17] for the association with landfills of urban waste.

## Conclusions

The present review highlighted that there is sufficient evidence of association between exposure to oil industry waste releasing high concentrations of hydrogen sulphide and acute symptoms. The evidence of causal relationship with hazardous waste was defined as limited for: liver, bladder, breast and testis cancers and non-Hodgkin lymphoma. Among non-neoplastic diseases, asthma was found to be related to hazardous waste with limited evidence. We evaluated as limited the evidence of the association between the exposure to hazardous waste and adverse birth outcomes, including low birth weight, pre-term birth, congenital anomalies overall and anomalies of the urogenital, connective and musculoskeletal systems. The evidence of a causal relationship was defined as inadequate for most other health outcomes.

The available evidence of the health effects of specific contaminants present in some hazardous waste corroborates our evaluation. In particular, drawing from the evidence described in the previous section: persistent organic pollutants, in particular benzene and those chlorinated, i.e. PCBs and dioxins (for liver, bladder and breast cancers, NHL and asthma), heavy metals, i.e. arsenic and its compounds (for NHL, liver and bladder cancers) and EDCs (for testis and breast cancers, CAs).

These data confirm that hazardous waste, if not suitably managed, might cause adverse health effects on populations living near the sites where they are dumped or processed. The contamination of different environmental matrices, including food, water, soil and air, represents a health risk for these populations. A strong contrast to illegal trade and the deployment of sound management practices is warranted as a priority – no further evidence is needed.

In several instances, however, further investigation is warranted to fill important knowledge gaps: in particular, population studies analysing different pathways of exposure, taking into account the characteristics of the site and the contaminants present in each waste site, might provide useful information. Acute respiratory diseases, diabetes and childhood neurological disorders are of particular interest, also in consideration of the strong indications that environmental exposures to EDCs and potential EDCs can cause cognitive and behavioural deficits in humans [40].

The results of our review, although not conclusive, provide indications that public health policies on hazardous waste management are urgently needed. International, national and local authorities should oppose and eliminate poor, outdated and illegal practices of waste disposal (including illegal transboundary trade), which still affect some communities in industrialized and middle-low income countries, and implement and enforce regulation. Compliance with the Basel Convention on the Control of Transboundary Movements of Hazardous Wastes and their Disposal is necessary to prevent high exposures and consequent health effects, particularly among the vulnerable and the poor [64].

## Additional files

Additional file 1: Table S1. Hazardous waste: general population mortality and morbidity [65–87] (XLSX 51 kb)

Additional file 2: Table S2. Hazardous waste: birth outcomes [88–105] (XLSX 30 kb)

Additional file 3: Table S3 Hazardous waste: acute effects (XLSX 12 kb)
